# Supplementary material for: All-fibre-coupled terahertz single-pixel imaging for biomedical applications
Source: Nat Commun. 2026 Jan 12;17:1571. doi: 10.1038/s41467-026-68290-x (PMC12901061; doi:10.1038/s41467-026-68290-x)
Supplement: Supplementary file 1 — Supplementary Information [file 41467_2026_68290_MOESM1_ESM.pdf]

Supplemental Information for:
All-fibre-coupled terahertz single-pixel imaging for biomedical applications

Sen Mou,[1] Rayko I. Stantchev,[1,2] Sonal Saxena,[3] Huiliang Ou,[1] Shreeya Rane,[1] Sophie L. Pain,[4,5] John D. Murphy,[4,5] Euan Hendry,[3] James Lloyd-Hughes,[1] and Emma Pickwell-MacPherson[1,*]

[1]*Department of Physics, University of Warwick, Coventry CV4 7AL, United Kingdom*

[2]*Department of Physics, National Sun Yat-Sen University, Kaohsiung, Taiwan*

[3]*Department of Physics and Astronomy, University of Exeter, Exeter, United Kingdom*

[4]*School of Engineering, University of Warwick, Coventry, CV4 7AL, United Kingdom*

[5]*School of Engineering, University of Birmingham, Edgbaston, Birmingham, B15 2TT, United Kingdom*

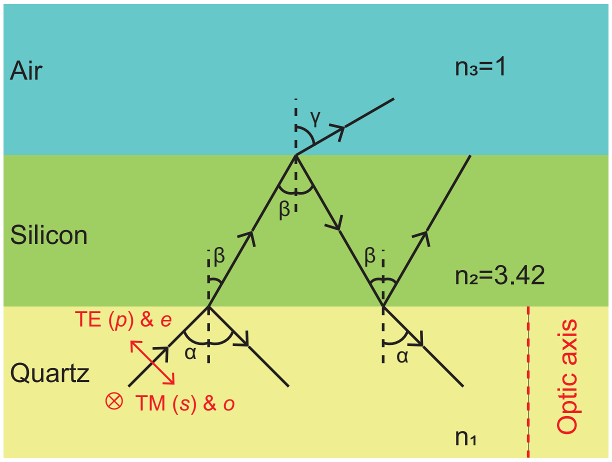

Supplementary Figure 1. THz light from the quartz prism enters the silicon wafer through the quartz-silicon interface, is reflected at the silicon-air interface, and returns to the prism through the silicon wafer and the quartz-silicon interface.

Effect of polarisation and birefringence on power transmission

As shown in Supplementary Fig. 1, Snell's law reads

$$n_1 \sin(\alpha) = n_2 \sin(\beta) = n_3 \sin(\gamma) \tag{1}$$

Supplementary Fig. 1 shows that the optical axis lies in the plane of incidence and is perpendicular to the material interfaces. The plane of incidence coincides with the plane formed by the wave vector and the optical axis. The polarisation of p-polarised light is in the plane of incidence. Thus, it also lies in the plane formed by the wave vector and the optic axis. As a result, p-polarised light is an extraordinary wave. The coincidence of p-polarised light and extraordinary wave ensures that the THz light experiences no ellipticity variation. The angle between the wave vector and the optical axis determines the extraordinary wave's refractive index. As shown in Supplementary Fig. 1, the angle between the wave vector and the optical axis is equal to the angle of incidence within the quartz. The extraordinary wave's refractive index can be expressed as[1]

$$n_{1,e}(\alpha) = 1/\sqrt{\left(\frac{\cos(\alpha)}{n_o}\right)^2 + \left(\frac{\sin(\alpha)}{n_e}\right)^2} \tag{2}$$

n_o and n_e are the refractive indices of the ordinary and extraordinary waves. The polarisation of s-polarised light is perpendicular to the plane of incidence and thus perpendicular to the optical axis. Consequently, the s-polarised light is an ordinary wave. Its refractive index is equal to n_o and is independent of the angle of incidence.

It is necessary to simultaneously account for the effects of polarisation and birefringence on Fresnel coefficients to calculate power transmittance. Assuming light is incident from a medium with refractive index n_i into a medium with refractive index n_j with an angle of incidence θ_i and refractive angle θ_j, the Fresnel reflection coefficient at the interface between the two materials for p- and s-polarised light can be expressed as follows:

$$r_{p,ij} = \frac{n_j \cos\theta_i - n_i \cos\theta_j}{n_j \cos\theta_i + n_i \cos\theta_j} \tag{3}$$

$$r_{s,ij} = \frac{n_i \cos\theta_i - n_j \cos\theta_j}{n_i \cos\theta_i + n_j \cos\theta_j} \tag{4}$$

Eqs. 3 and 4 can be rewritten as follows using Snell's law $n_i \sin\theta_i = n_j \sin\theta_j$.

$$r_{p,ij} = \frac{\tan(\theta_i - \theta_j)}{\tan(\theta_i + \theta_j)} \tag{5}$$

$$r_{s,ij} = -\frac{\sin(\theta_i - \theta_j)}{\sin(\theta_i + \theta_j)} \tag{6}$$

Supplementary Fig. 1 shows that THz transmits through the quartz-silicon interface, is reflected at the silicon-air interface and then returns to the quartz through the quartz-silicon interface again. The power transmittance of p-polarised light is $T_p = T_{p,12}R_{p,23}T_{p,21}$ with $T_{p,12} = 1 - R_{p,12}$ and $T_{p,21} = 1 - R_{p,21}$. $R_{p,ij} = |r_{p,ij}|^2$ is the power reflection coefficient for a p-polarised light from medium i to medium j. It can be found that $R_{p,12} = R_{p,21}$ from Eq. 5 and Supplementary Fig. 1, ensuring that if the light from quartz to silicon impinges on the interface at Brewster's angle, it will strike the interface again at Brewster's angle when the light comes back from silicon to quartz. The power transmittance of p-polarised light can be finally expressed as:

$$T_p = \left(1 - |r_{p,12}|^2\right)^2 |r_{p,23}|^2 \tag{7}$$

Similarly, the power transmittance of s-polarised light can be written as

$$T_s = \left(1 - |r_{s,12}|^2\right)^2 |r_{s,23}|^2 \tag{8}$$

When $\alpha + \beta = 90°$, i.e., $n_{1,e}\sin\alpha = n_2\sin\beta = n_2\cos\alpha$, the angle of incidence within the quartz is Brewster's angle at the quartz-silicon interface. One can calculate Brewster's angle by substituting Eq. 2 into $n_{1,e}\sin\alpha = n_2\cos\alpha$.

$$\alpha_{bst,12} = \arccos\left(\sqrt{\frac{\sqrt{4n_o^2n_2^2n_e^4 + [n_o^2(n_2^2 - n_e^2)]^2} - n_o^2(n_2^2 + n_e^2)}{2n_2^2(n_e^2 - n_o^2)}}\right) \tag{9}$$

Another Brewster's angle appears at the silicon-air interface when $\beta + \gamma = 90°$, i.e., $n_2\sin\beta = n_3\sin\gamma = n_3\cos\beta$. The angle of incidence in silicon for Brewster's at the silicon-air interface reads

$$\beta_{bst,23} = \arctan\left(\frac{n_3}{n_2}\right) \tag{10}$$

To calculate the angle of incidence for this Brewster's angle, we should express α using β. Substituting Eq. 2 into the expression $n_{1,e}\sin(\alpha) = n_2\sin(\beta)$, the following formula can be obtained.

$$\alpha = \arctan\left(\frac{n_e n_2 \sin\beta}{n_o\sqrt{n_e^2 - (n_2\sin\beta)^2}}\right) \tag{11}$$

The angle of incidence in quartz ($\alpha_{bst,23}$) for Brewster's angle at the silicon-air interface can be calculated by substituting Eq. 10 into Eq. 11.

Supplementary Table 1. The angles of incidence in quartz for Brewster's angles at quartz-silicon and silicon-air interfaces and the critical angle at the silicon-air interface

p-polarised			s-polarised
Brewster (Si2air)	Critical (Si2air)	Brewster (quartz2Si)	Critical (Si2air)
26.9°	28.1°	57.9°	28.3°

The p-polarised light can also undergo total reflection at the silicon-air interface. To calculate the angle of incidence in quartz ($\alpha_{c,p}$) for the critical angle at the silicon-air interface, we can set $\gamma = 90°$, leading to $n_{1,e}\sin(\alpha) = n_3$. Substituting Eq. 2 to this equation, $\alpha_{c,p}$ can be written as:

$$\alpha_{c,p} = \arctan\left(\frac{n_e}{n_o\sqrt{n_e^2 - 1}}\right) \tag{12}$$

A s-polarised light is an ordinary wave, which only experiences a critical angle when α varies from 0° to 90°. Similarly, Eq. 1 gives rise to $n_o\sin(\alpha) = n_3$. The angle of incidence in quartz for the critical angle at the silicon-air interface reads:

$$\alpha_{c,s} = \arcsin\left(\frac{n_3}{n_o}\right) \tag{13}$$

Substituting the known parameters $n_o = 2.11$, $n_e = 2.16$ [2], $n_2 = 3.42$[3] and $n_3 = 1$ into the above formulas, we can calculate the angles of incidence in quartz for Brewster's angles of p-polarised THz light at quartz-silicon and silicon-air interfaces and the critical angles of p- and s-polarised THz light at the silicon-air interface. The results are presented in Supplementary Table 1.

Design and optimisation of fibre projection system

We report the implementation of a fibre-coupled spatial light modulation system for THz imaging, employing a digital micromirror device (DMD) to encode and project dynamic patterns onto a silicon surface. One of the key challenges in fully fibre-coupling the THz imaging setup is transporting these patterned images from the DMD via an optical fibre to the detection plane. To maximise laser power transmission while preserving image resolution, a multi-core image fibre with a large core diameter of 1.4 mm and comprising 100,000 picture elements is utilised. A 10× microscope objective lens (MO1), selected for its entrance pupil diameter and numerical aperture, facilitates efficient coupling of the patterned image from the DMD to the input facet of the optical fibre over a short working distance. The area of

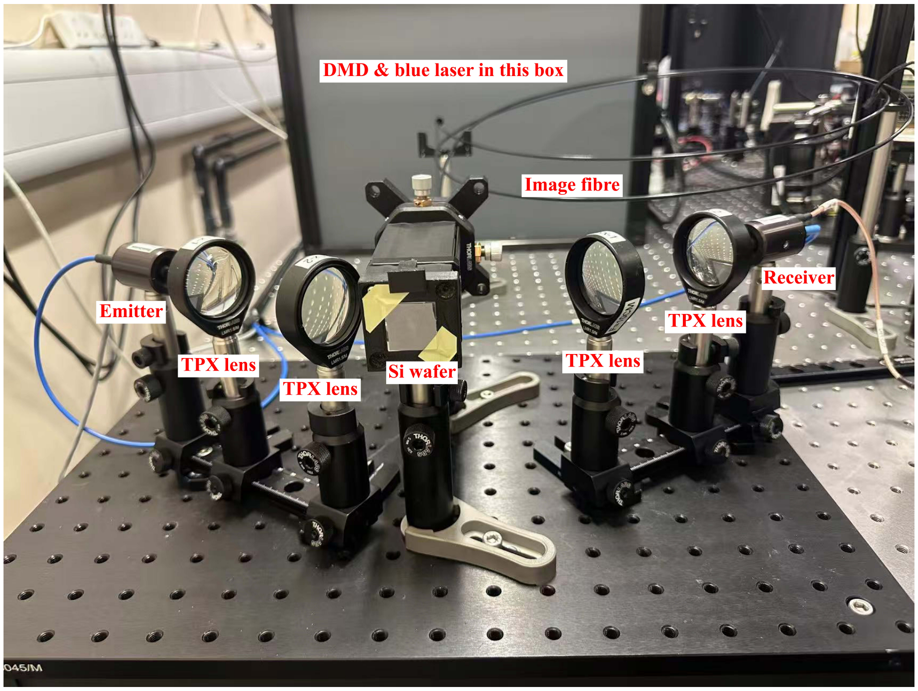

Supplementary Figure 2. Photograph of the all-fibre-coupled THz ATR single-pixel imaging system. The silicon wafer is attached to the surface of the quartz prism.

the DMD used for pattern projection is chosen for optimal coupling, matching the image size to the area of the fibre bundle. The numerical aperture of the lens is also approximately matched to the acceptance angle of the fibres. After the fibre bundle, the output from the optical fibre is re-imaged onto the silicon surface using a second, matched microscope objective lens (MO2). Note, the imaging fibre bundle can only transfer spatial-intensity information (and not phase), which needs to be considered when designing such an imaging system.

Laser power losses inevitably occur at each stage of the coupling process. The micromirrors of the DMD behave as a blazed diffraction grating, generating multiple diffracted orders. Additional losses arise from specular reflection off the DMD protective cover and from light scattering within the gaps separating the individual micromirrors. Furthermore, even with efficient hexagonal packing, the imaging fibre bundle inherently possesses cladding regions surrounding each picture element, creating non-transmissive areas. The cumulative effect of Fresnel reflections at all optical interfaces throughout the system, coupled with intrinsic transmission losses in the optical fibre, leads to a non-negligible reduction in the delivered optical power. The measured laser power transmission values of the optical components are

Supplementary Table 2. Transmission of the optical components in the 450nm fibre-coupling setup.

Optical component	DMD	MO1 & MO2	Fibre	Prism	Total
Transmission	52%	82%	66%	91%	21%

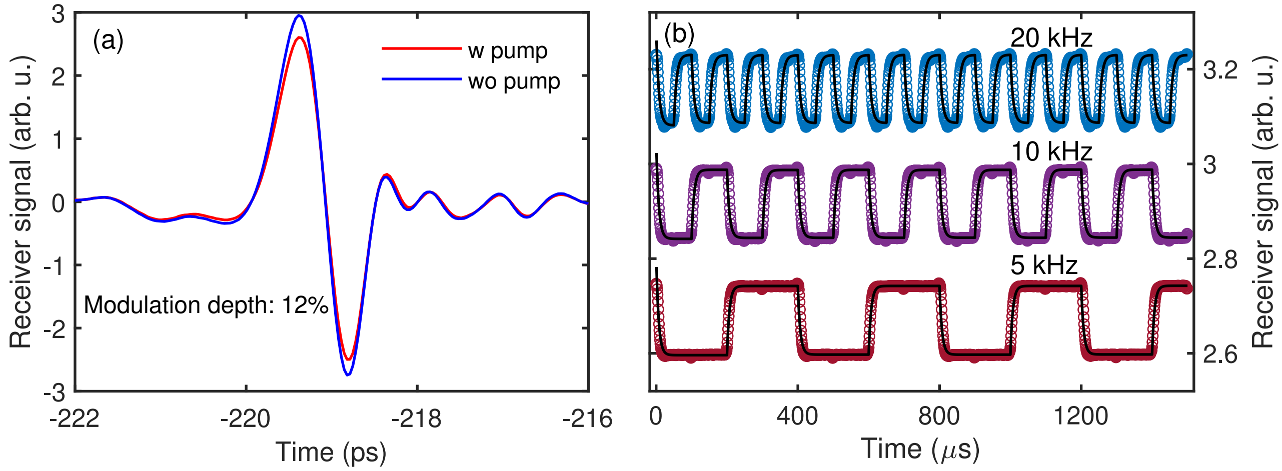

Supplementary Figure 3. Photo modulation. (a) Receiver signals with and without photo modulation. Modulation depth is 12%. (b) THz modulation at receiver signal peak with full-on and -off masks with DMD switching rate equal to 5, 10 and 20 kHz. Symbols stand for experimental data, and solid lines are fitting results. The data in (b) are vertically shifted for clarity.

summarised in Supplementary Table 2. Supplementary Fig. 2 shows a photograph of the system.

While the entire optical system can be aligned to maximise laser power transmission to the silicon surface, it is crucial to recognise that maximising power alone does not guarantee optimal image resolution and accurate image dimensions. To ensure a sharp, high-fidelity image in the plane of the silicon surface, only the first diffracted order from the DMD is collected and re-imaged. This is achieved by precisely optimising the spacing between the optical fibre and both microscope objectives, ensuring that the angular acceptance of the optics is well-matched to the desired diffraction order and that the encoded pattern is accurately relayed onto the silicon surface.

Modulation depth

Supplementary Fig. 3a compares receiver signals with and without photo modulation, showing that the optical pump reduces THz intensity. We define the modulation depth as

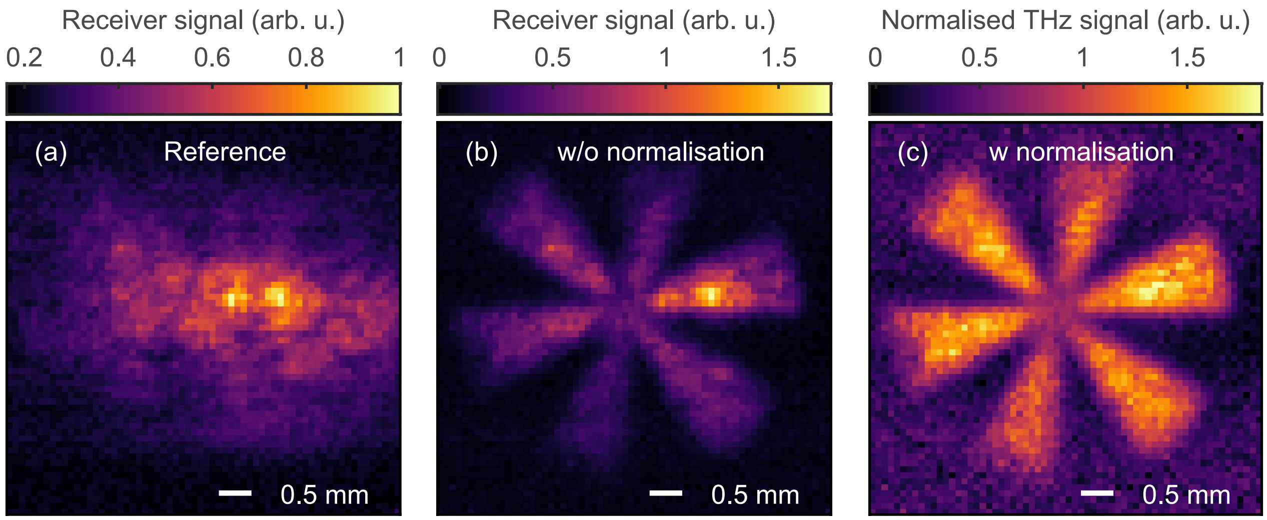

Supplementary Figure 4. Image normalisation. (a) Reference image normalised to its maximum value $I_{\mathrm{ref}}^{\mathrm{norm}}(m,n)$ (see Supplementary Eq. 15) obtained with a bare silicon wafer, showing the profile of the THz beam. (b) Time-domain image of cartwheel sample at THz peak without normalisation $I_{\mathrm{smp}}(m,n)$. (c) Normalised Image of cartwheel sample at peak $I_{norm}(m,n)$ (see Supplementary Eq. 16).

the ratio between the difference peak-to-peak value and the peak-to-peak value of the THz signal without a pump. The calculated modulation depth is 12%. Supplementary Fig. 3b compares modulation signals at the peak of the receiver signals with full-on and -off DMD masks with switching rates equal to 5, 10, and 20 kHz. The symbols are experimental data, and solid lines represent fitting results. The experimental data of 50 measurements are globally fitted with an exponential function to extract carrier lifetime using a least-squares fitting algorithm [4]. The extracted lifetime is 7 μs. The data shown in Supplementary Fig. 3b are the averages of the 50 measurements.

Image normalisation

The time-domain images in Figs. 3a, 3b, 4, 5a and 6 in the main text are normalised to a THz beam reference image. For Fig. 3 of the main text, we normalised the time-domain images at all the time points with the same constant reference image, then Fourier transformed the normalised time-domain THz signal at each pixel to obtain its spectrum. The spectra of all the pixels give the frequency-domain images. Fig. 3c of the main text is the frequency-domain image at the spectrum peak. We note that Fig. 5b and 5c of the main text are images of $|\hat{H}(\omega)|$ and $\Delta\phi(\omega)$, which are calculated with Eq. 2 rather than by

normalising with a constant reference image.

The reference image (Supplementary Fig. 4a) accounts for the profile of the THz beam, which was acquired using a bare silicon wafer under identical experimental conditions. We adopted the summed frequency-domain amplitude image as the reference image, shown below:

$$I_{\text{ref}}\,(m,n) = \sum_{f} I_{\text{ref}}\,(m,n,f) \tag{14}$$

$I_{\text{ref}}(m,n,f)$ is the frequency-domain amplitude image at frequency f. m and n represent the number of horizontal and vertical pixels. As the summation increases, the amplitude of the resulting image becomes a finite, huge number; thus, we normalise it to its maximum value.

$$I_{\text{ref}}^{\text{norm}}(m,n) = \frac{I_{\text{ref}}\,(m,n)}{max(I_{\text{ref}}\,(m,n))} \tag{15}$$

The final results (Figs. 3a, 3b, 4, 5a and 6 in the main text) are acquired by normalising the time-domain sample images ($I_{\text{smp}}\,(m,n)$) with the normalised reference image ($I_{\text{ref}}^{\text{norm}}(m,n)$).

$$I_{norm}(m,n) = \frac{I_{\text{smp}}(m,n)}{I_{\text{ref}}^{\text{norm}}(m,n)} \tag{16}$$

An example of normalisation demonstrating the improvement of image quality is shown in Supplementary Fig. 4. Supplementary Fig. 4b displays the time-domain image of the cartwheel at the peak of the THz signal, revealing an inhomogeneous intensity distribution across the cartwheel arms. Supplementary Fig. 4c presents the normalised cartwheel image, illustrating how normalisation effectively mitigates the adverse effects of inhomogeneous THz and optical pump profiles on the image quality.

Resolution, signal-to-noise ratio and image contrast

To estimate the resolution, contrast, and signal-to-noise ratio (SNR), we fabricated a knife-edge sample, which was obtained by depositing a layer of gold to cover half of a silicon wafer. We first obtained THz images with a bare silicon wafer as a reference image. Then we imaged the knife-edge sample. The images of the knife-edge sample are normalised with the reference image to mitigate the adverse inhomogeneity effect arising from the THz beam and the blue light. The normalised frequency-domain images at the spectrum peak (0.63 THz) are shown in the first row of Supplementary Fig. 5. Profiles of lines perpendicular to the sharp edge are employed to estimate the resolution. Example lines are shown in the first row (Supplementary Fig. 5a-d) while the corresponding profiles are plotted in the

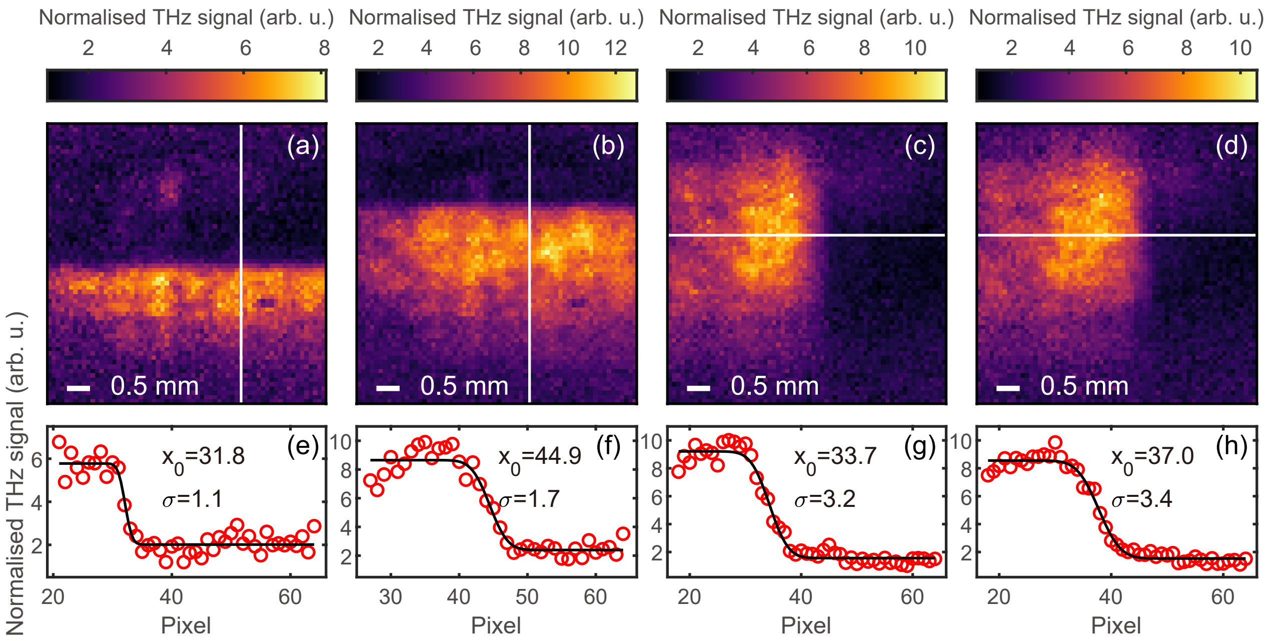

Supplementary Figure 5. Image resolution estimation. Images of a horizontally aligned knife-edge sample at different positions (a & b). Images of a vertically aligned knife-edge sample at different positions (c & d). Images in the first row are frequency-domain ones at the spectrum peak of 0.63 THz. The second row (e-h) shows the profiles of the white lines in the first row. The red circles are the experimental data, and the solid lines denote the fitting result in e-f. Fitting parameters x_0 and σ are also presented in each panel.

second row (Supplementary Fig. 5e-f). The symbols in Supplementary Fig. 5e-f represent the experimental result, and the black solid lines are the fitting results with a Gaussian edge spread function (ESF).

$$\mathrm{ESF(x)} = a + \frac{b}{2}\left[1 - \mathrm{erf}\left(\frac{x - x_0}{\sqrt{2}\sigma}\right)\right] \tag{17}$$

Where a stands for the baseline offset, b represents the step height, x_0 denotes the location of the edge, σ is the blur parameter related to resolution, and erf is the error function. The distance related to the fitted 10–90% step height is assumed to be the resolution. The estimated resolution is 360 μm for a horizontally aligned edge, and 660 μm for a vertically aligned edge. The different resolutions for the horizontally and vertically aligned edges may arise from the effect of THz polarisation and the reflection configuration. Due to the reflection configuration, the THz light obliquely enters and exits the silicon wafer. The enter

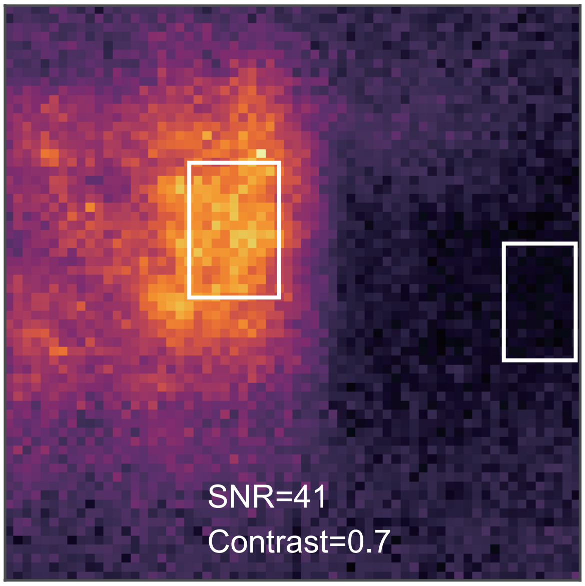

Supplementary Figure 6. Signal-to-noise ratio and image contrast. The brightest and darkest regions are applied to estimate the Signal-to-noise ratio and image contrast.

and exit positions exhibit a horizontal side shift (see Supplementary Fig. 1), which is likely to worsen the horizontal resolution.

We also applied the image of the knife-edge sample to estimate the SNR. The brightest and darkest regions in the image are selected as shown in Supplementary Fig. 6. The ratio of the average value of the brightest region to the standard deviation of the darkest region is assumed to be the SNR[4]. The estimated SNR is 41. The brightest and darkest regions are also applied to estimate the contrast. The contrast is assumed to be the ratio of the difference of the average values of these two regions to the sum of the average values. The estimated contrast value is 0.7.

Real-time *in vivo* imaging

In the video recording the real-time *in vivo* experimental measurement of the scab, the left window shows the result of a web-camera monitoring the measurement process and the right window displays the THz image result. The raw and normalised THz image of the scab and THz beam profile are also shown in the video.

* e.macpherson@warwick.ac.uk

[1] Z. Xu, S. Mou, L. Tomarchio, A. D'Arco, K. Guo, M. Petrarca, and S. Lupi, Phase-matching effect on the second harmonic and terahertz generations in β-barium borate, Opt. Laser Technol. **167**, 109764 (2023).

[2] E. Castro-Camus, J. Lloyd-Hughes, M. D. Fraser, H. H. Tan, C. Jagadish, and M. B. Johnston, Detecting the full polarization state of terahertz transients, in *Terahertz and Gigahertz Electronics and Photonics V*, Vol. 6120, edited by R. J. Hwu and K. J. Linden, International Society for Optics and Photonics (SPIE, 2006) p. 61200Q.

[3] S. Wu, Y. Yuan, G. Li, Y. Zhu, and L. Chen, Increase the sensitivity of terahertz liquid detection using a triple attenuated total reflection probe, Opt. Express **33**, 5021 (2025).

[4] R. I. Stantchev, X. Yu, T. Blu, and E. Pickwell-MacPherson, Real-time terahertz imaging with a single-pixel detector, Nat. Commun. **11**, 2535 (2020).
