# Supplementary material for: All-fibre-coupled terahertz single-pixel imaging for biomedical applications
Source: Nat Commun. 2026 Jan 12;17:1571. doi: 10.1038/s41467-026-68290-x (PMC12901061; doi:10.1038/s41467-026-68290-x)
Supplement: Supplementary file 2 — Description of Additional Supplementary Files [file 41467_2026_68290_MOESM2_ESM.pdf]

Description of Additional Supplementary Files

Supplementary Movie 1: The supplementary movie shows the in vivo imaging of a scab on an elbow, making a live comparison of the images of the THz beam without normalization, scab without normalization, scab with normalisation and THz beam with normalisation. The image of scab with normalisation clearly illustrates the region of the scab.
